# Compact integrated self-multiplexing antenna for sub-6 GHz and millimeter wave 5G frequency spectrum

**DOI:** 10.1038/s41598-026-35031-5

**Published:** 2026-01-16

**Authors:** Gunjan Srivastava, Amit Kumar, Sandeep Rana, Vimal Kumar, Akhilesh Mohan, Sachin Kumar, Tanweer Ali

**Affiliations:** 1 Department of Electronics and Communication Engineering, Graphic Era (Deemed to be) University, Dehradun, 248002 India; 2 Department of Electronics and Communication Engineering, Indian Institute of Technology Roorkee, Roorkee, 247667 India; 3 Department of Electronics and Communication Engineering, G.B. Pant Institute of Engineering & Technology, Pauri Garhwal, 246194 India; 4 Department of Electronics and Communication Engineering, Galgotias College of Engineering and Technology, Greater Noida, 201310 India; 5 Manipal Institute of Technology, Manipal Academy of Higher Education, Manipal, India

**Keywords:** Engineering, Optics and photonics, Physics

## Abstract

This paper presents a compact integrated self-multiplexing antenna capable of operating across both the sub-6 GHz and millimeter-wave bands of the 5G frequency spectrum. The sub-6 GHz unit elements are alternately arranged with different structural dimensions to achieve eight distinct operating frequencies within the sub-6 GHz spectrum. Between each pair of sub-6 GHz unit elements, millimeter-wave unit elements of varying dimensions are incorporated to generate radiations at eight distinct frequencies in the millimeter-wave band. The first eight ports (P_1_–P_8_) operate at eight distinct frequencies of sub-6 GHz spectrum, whereas the remaining eight ports (P_9_–P_16_) are designed to radiate at eight distinct millimeter-wave frequencies. Electromagnetic waves in the sub-6 GHz spectrum are obtained by means of exciting TE_110_ mode in the modified eighth-mode substrate integrated waveguide (EMSIW) cavity resonators, while millimeter-wave radiation is achieved through the hybrid TE_730_ and TE_750_ modes of the EMSIW cavity resonators. The inter-port isolations better than 40 dB and 20 dB are obtained across the sub-6 GHz and millimeter-wave spectrum, respectively. This integrated placement of millimeter-wave elements efficiently utilizes the available substrate area while maintaining high inter-port isolation across the entire frequency spectrum. The design antenna system has an overall footprint of 0.425$\:{\boldsymbol{\lambda\:}}_{\boldsymbol{g}}^{2}$, where $\:{\boldsymbol{\lambda\:}}_{\boldsymbol{g}}$ corresponds to the guided wavelength at the lowest operating frequency. Owing to its compact dimensions, simple integrated design and excellent performance, the proposed self-multiplexing antenna emerges as a promising candidate for multiband communication systems operating in sub-6 GHz and millimeter-wave 5G spectrum.

## Introduction

With the rapid advancement in fifth-generation (5G) communication technology, there is a great demand for compact and multifunctional antennas^1,2^. These multifunctional antennas support multiple operations within compact dimensions. To achieve multifunctional operation, either several single band antennas or a single multiband antenna is typically required. The former one requires external multiplexer to select a particular frequency band, which in turn increases the system complexity and overall footprint. While the latter offers multiband operation with single excitation, but they often face demerits such as reduced efficiency, poor inter-band isolation, and complex design trade-offs^3–5^. On the other hand, self-multiplexing antennas are increasingly being employed nowadays, as they provide multiband operation by assigning each individual port to a specific frequency channel.

Substrate integrated waveguide (SIW) technology has gained significant attention for the design of self-multiplexing antennas, owing to its inherent merits of low cost, compact profile, low loss, high inter-port isolation, unidirectional radiation behaviour, and seamless integration with planar circuits^6^. Full-mode SIW (FMSIW) cavity resonators and their miniaturized versions, such as half-mode SIW (HMSIW)^7,8^, quarter-mode SIW (QMSIW)^9,10^ and eighth-mode SIW (EMSIW) cavity resonators^11,12^, have been extensively employed in the design of self-multiplexing antennas operating in two^13–16^, three^17–20^, four^21–24^, five^25^, six^26–28^ and eight^29,30^ distinct frequency bands of the microwave spectrum. The millimeter-wave spectrum has recently attracted significant attention in 5G communication systems due to its wide bandwidth, ultra-high data rates, and substantially improved channel capacity^31,32^. Two SIW based self-multiplexing antennas are designed and developed supporting four^31^ and eight^32^ distinct millimeter-wave bands. As the microwave and millimeter-wave spectrums offer complementary benefits, the microwave spectrum supports long-distance communication with limited bandwidth, whereas the millimeter-wave spectrum enables high data rates but is restricted to short-distance communication due to high propagation losses.

Owing to their complementary benefits, a few self-multiplexing antennas capable of operating simultaneously in both microwave and millimeter-wave frequency bands have been reported in recent years^33–37^. A dual-band self-multiplexing antenna utilizing HMSIW cavity resonators is reported^34^, demonstrating operation at 5.4 GHz in the microwave band and 29 GHz in the millimeter-wave band. Another design of a self-multiplexing antenna^35^ has been reported, which operates in the microwave band at 5.25 GHz and in the millimeter-wave band at 28.85 GHz and 31.9 GHz, thereby demonstrating simultaneous dual-spectrum operation. Further, two self-multiplexing antennas operating in two microwave bands and two millimeter-wave bands also have been reported^36,37^. Nowadays, wireless communication systems demand a greater number of channels to accommodate the ever-increasing data traffic and to ensure the efficient utilization of the limited available spectrum. While these designs demonstrate the feasibility of achieving dual-spectrum operation, but they are limited in terms of the number of supported frequency channels. The utilization of a larger number of frequency bands across both the sub-6 GHz and millimeter-wave spectrum is highly desirable for future-generation communication systems, as it enables enhanced spectrum efficiency and supports diverse service requirements. As the number of operating bands increases, maintaining sufficient inter-port isolation along with achieving high-gain characteristics becomes increasingly difficult. Therefore, designing an integrated self-multiplexing antenna that can support a large number of bands across both the sub-6 GHz and millimeter-wave spectra remains a significant challenge for researchers.

Motivated by these limitations, this work proposes a 16-port integrated self-multiplexing antenna capable of simultaneous operation across both the sub-6 GHz and millimeter-wave frequency bands, thereby offering enhanced functionality within a compact structure. The first eight ports of the designed antenna operate at sub-6 GHz frequency spectrum, while the other eight ports at millimeter-wave spectrum. Ports P_1_-P_8_ radiate electromagnetic waves in the sub-6 GHz spectrum by exciting the TE_110_ mode of the modified EMSIW cavity resonators. While Ports P_9_-P_16_ radiate millimeter-wave through the hybrid TE_730_ and TE_750_ modes of EMSIW cavity resonators. In the sub-6 GHz spectrum, the self-multiplexing operation is realized at 2.1, 2.35, 2.75, 3.25, 3.85, 4.15, 4.50, and 4.90 GHz, whereas in the millimeter-wave spectrum, it operates at 28.75, 29.25, 29.55, 30, 30.5, 31, 31.65, and 32.50 GHz. The inter-port isolations better than 40 dB and 20 dB are obtained across the sub-6 GHz and millimeter wave spectrum, respectively.

To emphasize the merits of the proposed design, its performance is compared with other self-multiplexing antennas reported in the literature that operate within the sub-6 GHz and/or millimeter-wave spectrums in Table 1. Compared to ref.^20–30^, the proposed self-multiplexing antenna supports communication over a larger number of bands in the microwave spectrum, including the sub-6 GHz band. The self-multiplexing antennas presented in ref.^31,32^ offer radiation in four and eight distinct bands of the millimeter-wave spectrum. Integrated self-multiplexing antennas capable of simultaneous operation in both microwave and millimeter-wave frequency bands have been reported^33–37^. All of them operate simultaneously in either one or two microwave and millimeter-wave frequency bands. The utilization of a larger number of frequency bands across both the sub-6 GHz and millimeter-wave spectrum is highly desirable for future generation communication systems, as it enables enhanced spectrum efficiency and supports diverse service requirements. With its capability of simultaneous operation in sub-6 GHz and millimeter-wave bands, the proposed 16-port integrated antenna can be a potential candidate for multi-band communication systems.

**Table 1:** Comparison of the proposed antenna with reported sub-6 GHz and millimeter wave self-multiplexing antennas.

Ref.	No. of Ports (LB, UB)	Freq. in LB (in GHz)	Freq. in UB (in GHz)	Max. Gain LB/UB (dBi)	Isolation (LB/UB) (dB)
^20^	3 (LB), --	2.55, 3.99, 5.2	--	5.2, --	33, --
^21^	4 (LB), --	2.29, 2.86, 3.11, 3.44	--	5, --	30, --
^22^	4 (LB), --	8.19, 8.8, 9.7, 11	--	7.47, --	36, --
^23^	4 (LB), --	2.45, 3.5, 4.9, 5.4	--	5.97, --	13, --
^24^	4 (LB), --	4.35, 5.35, 5.9, 6.75	--	6.12, --	22, --
^25^	5 (LB), --	2.29, 2.98, 3.65, 4.37, 5.08	--	5.70, --	--, 30
^26^	6 (LB), --	5.33, 5.76, 6.31, 6.86, 7.34, 7.8	--	6.60, --	--, 30
^27^	6 (LB), --	2.29, 2.96, 4.30, 5.0, 5.61, 6.18	--	5.46, --	--, 30
^28^	6 (LB), --	4, 5.8, 6.6, 7.8, 9.8,10.68	--	5.43, --	--, 27
^29^	8 (LB), --	5.15, 5.67, 6.18, 6.6, 7.18, 7.85, 8.25, 8.85	--	4.14, --	--, 15
^30^	8 (LB), --	3.35, 3.58, 3.73, 3.91, 4.09, 4.32, 4.61, 4.96	--	4.74, --	35, 35
^31^	--, 4 (UB)	--	24.25, 26.5, 27.5, 29.5	--, 4.48	--, 20
^32^	--, 8 (UB)	--	25.8, 27.5, 29.5, 31, 35.6, 36.8, 38.2, 39.7	--, 12.2	--, 29
^33^	1 (LB), 1 (UB)	5.2	24	3.93, 6.32	40, 30
^34^	1 (LB), 1 (UB	5.4	29	4, 8.85	34, 28
^35^	1 (LB), 2 (UB)	5.25	28.85, 31.9	5.1, 8.3	33, 33
^36^	2 (LB), 2 (UB)	5.8, 7.4	28, 38	5.2, 8.3	30, 20
^37^	2 (LB), 2 (UB)	4.8, 5.4	28, 30	5.2, 8.7	30, 22
Prop.	8 (LB), 8 (UB)	2.10, 2.35, 2.75, 3.25, 3.85, 4.15, 4.50, 4.85	28.75, 29.25, 29.50, 29.95, 30.50, 31, 31.65, 32.50	5.1, 10.55	40, 20

LB: Lower band, UB: Upper band.

The salient features of the proposed integrated 16-port self-multiplexing antenna are as follows:

- The proposed 16-port integrated antenna provides simultaneous radiation coverage across sixteen distinct bands in both the sub-6 GHz and millimeter-wave spectrum.
- The 8-port sub-6 GHz self-multiplexing antenna and the 8-port millimeter-wave self-multiplexing antenna are co-designed on the same substrate, achieving inter-port isolation of 40 dB and 20 dB, respectively.
- The designed antenna can be re-designed to any other frequency which lies in 2.0–6.0 GHz in sub-6 GHz spectrum and 28.6–32.7 GHz in millimeter-wave spectrum.
- The proposed antenna finds its application in 5G NR communication systems, particularly within the FR1 and FR2 frequency bands.

To the best of authors’ knowledge, this is the first demonstration of simultaneous self-multiplexing operations in sixteen distinct frequencies, covering both sub-6 GHz and millimeter-wave bands, while maintaining good isolation.

## Antenna configuration

**Fig. 1:**
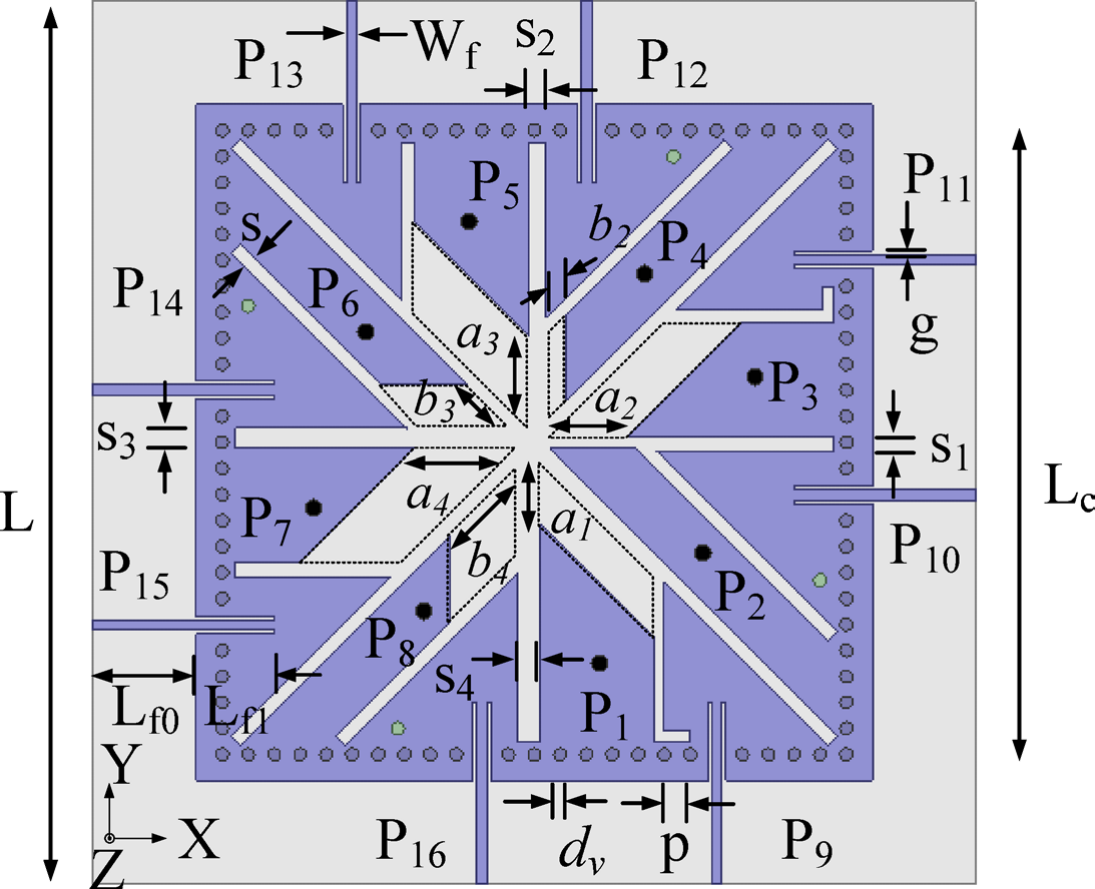
Geometrical configuration of the proposed 16-port integrated self-multiplexing antenna [*L* = 68, *L*_*c*_ = 48, *L*_*f*0_ = 8.0, *L*_*f*1_ = 6.0, *W*_*f*_ = 0.77, *s* = 1, *s*_1_ = 1.2, *s*_2_ = 1.4, *s*_3_ = 1.6, *s*_4_ = 1.8, *a*_1_ = 6.2, *a*_2_ = 7.2, *a*_3_ = 8.2, *a*_4_ = 9.2, *b*_2_ = 1.6, *b*_3_ = 3.4, *b*_4_ = 5.2, *g* = 0.3, *p* = 2, *d*_*v*_ = 1 (all dimensions are in mm)].

Figure 1 illustrates the geometrical configuration of the proposed 16-port integrated self-multiplexing antenna for sub-6 GHz and millimeter wave 5G frequency spectrum. The self-multiplexing antenna is implemented on the RT/Duroid 5880 substrate with a dielectric constant of 2.2 and a loss tangent of 0.0008. In this design, the substrate thickness is chosen as 0.254 mm. The antenna elements of the proposed integrated self-multiplexing antenna are embedded within a square SIW cavity. The SIW cavity, with dimensions *L*_*c*_*× L*_*c*_​, is realized by introducing an array of metallic vias of diameter *d*_*v*_ and spacing *p* in the dielectric substrate. To minimize the field leakage through the cavity sidewalls^6^, the via parameters are chosen as *d*_*v*_ = 1 mm and *p* = 2 mm.

The integrated self-multiplexing antenna is constructed using the EMSIW cavity resonator as its basic element. Eight coaxial connectors are employed to excite the TE_110_ mode of EMSIW cavities for self-multiplexing operation in sub-6 GHz, whereas eight inset fed microstrip lines are used to excite hybrid TE_730_ and TE_750_ modes of the EMSIW cavities resonators for millimeter-wave. The basic elements corresponding to the sub-6 GHz and millimeter-wave spectrums are interleaved within the square SIW cavity to ensure efficient utilization of the limited space. This arrangement also contributes to achieving high inter-port isolation.

**Fig. 2:**
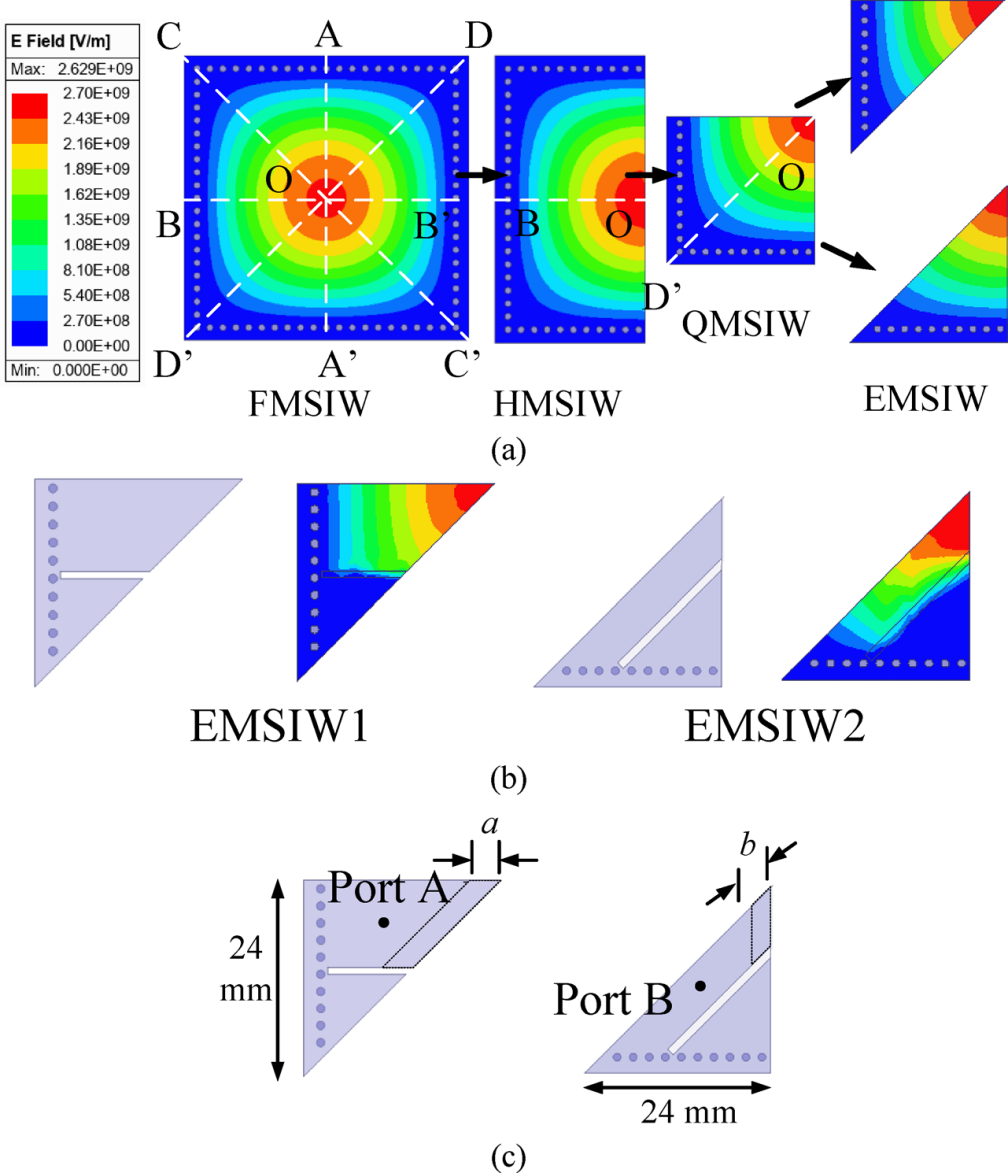
**a** Development stages of the EMSIW cavity resonator, **b** EMSIW1 and EMSIW2 cavity resonators, **c** Coax-fed EMSIW cavity resonators.

**Fig. 3:**
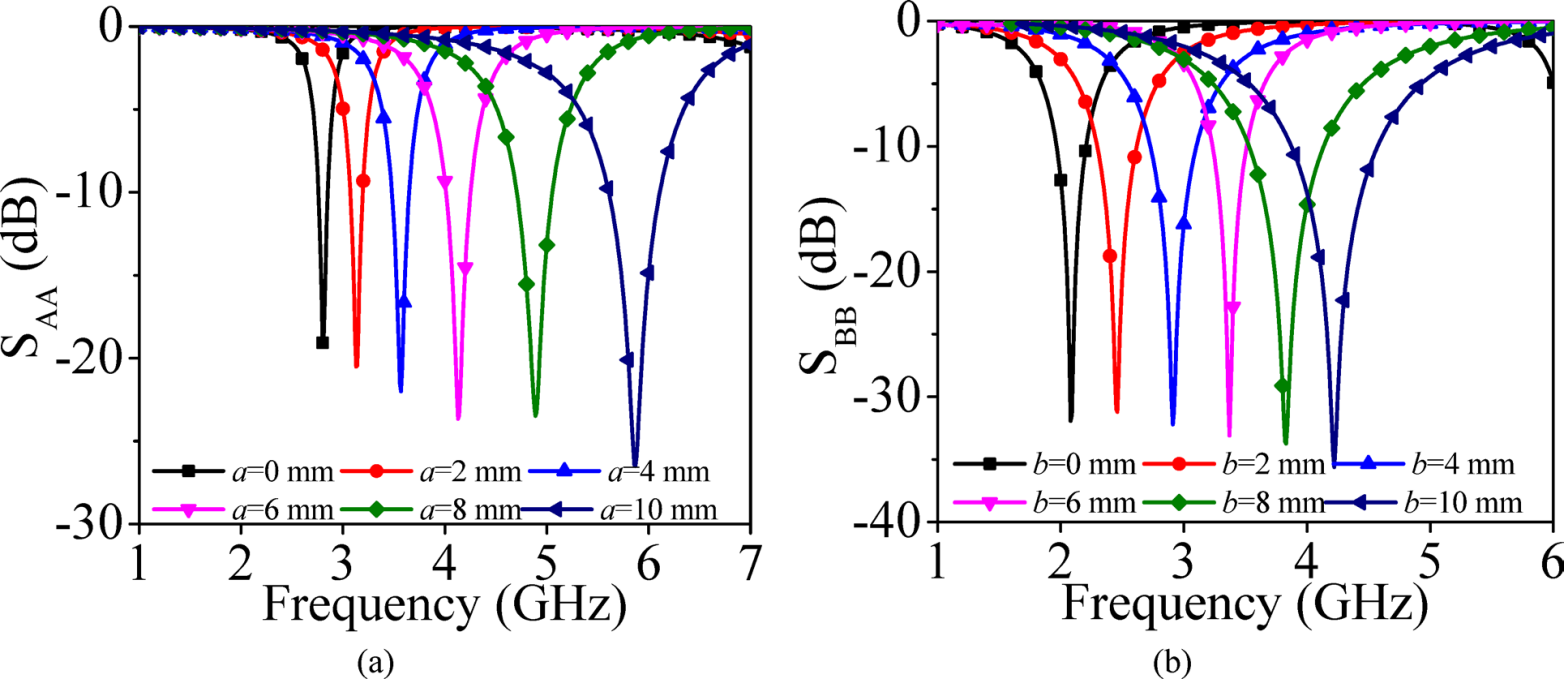
Simulated S-parameters for different values of **a** design parameter *a* in the coax-fed EMSIW1 cavity, **b** design parameter *b* in the coax-fed EMSIW2 cavity (corresponding to the structures shown in Fig. 2c).

## Design methodology

In this section, the detailed design methodology of the proposed 16-port integrated self-multiplexing antenna for sub-6 GHz and millimeter-wave frequency spectrum is presented.

### Basic element for sub-6 GHz self-multiplexing operation

Figure 2 illustrates the electric field distribution of the TE_110_ mode in the square SIW cavity. Since the fields in a FMSIW cavity are symmetrical across the planes AA’ and BB’, the cavity can be divided into two equal halves by applying the magnetic wall concept, resulting in a HMSIW cavity. This magnetic wall concept is further applied on HMSIW cavity to subsequently obtain QMSIW and EMSIW cavity resonators. These bifurcations in the cavity resonators not only preserve the modal characteristics of the TE_110_ mode but also contribute to size reduction. The basic EMSIW cavity resonator is further modified by introducing rectangular slots, resulting in the EMSIW1 and EMSIW2 cavity resonators (Fig. 2b). In the EMSIW1 cavity resonator, a horizontal rectangular slot is incorporated, whereas in the EMSIW2 cavity resonator, a slanted rectangular slot is introduced. These rectangular slots provide a pathway for the placement of millimeter-wave elements and contribute to size miniaturization.

Figure 2c illustrates the coaxial fed EMSIW1 and EMSIW2 basic elements. The operating frequencies of these basic EMSIW elements can be altered with varying dimension *a* in EMSIW1 and *b* in EMSIW2 elements, respectively, which is required for self-multiplexing operations.

Figure 3 presents the simulated S_AA_-parameters of the coaxially fed EMSIW1 cavity resonators for different values of the design parameter *a* and the simulated S_BB_-parameters of the coaxially fed EMSIW2 cavity resonators for different values of the design parameter *b*. Increasing the value of *a* reduces the size of the EMSIW1 cavity resonator, while increasing *b* decreases the effective size of the EMSIW2 cavity resonator; in both cases, the operating frequency shifts to a higher value. The coax-fed EMSIW1 cavity resonators are employed for self-multiplexing in the upper sub-6 GHz band, whereas the coax-fed EMSIW2 cavity resonators are utilized in the lower sub-6 GHz band.

**Fig. 4:**
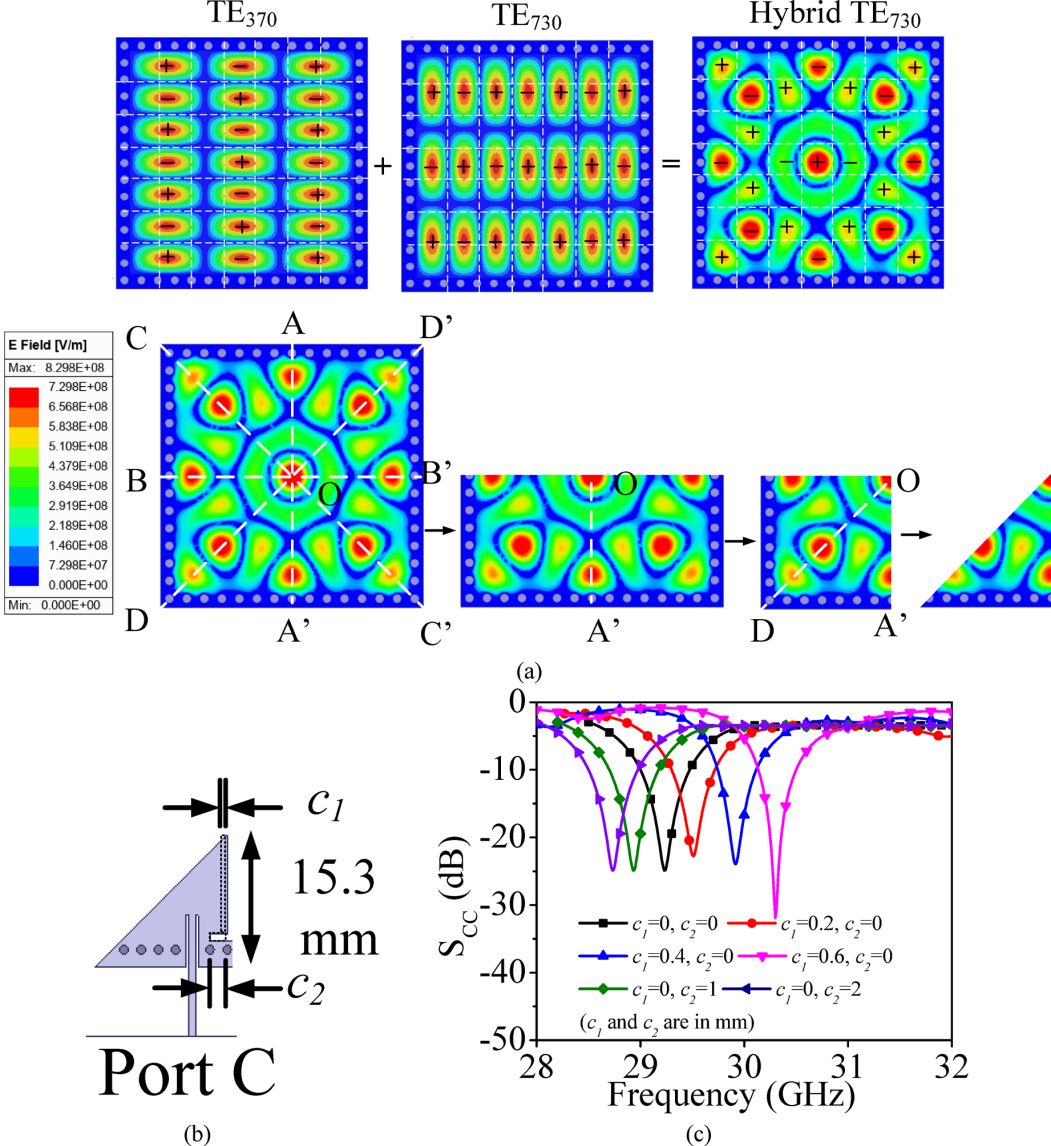
**a** Development stages of the EMSIW cavity resonator utilizing hybrid TE_730_ mode, **b** Microstrip-fed EMSIW cavity resonator, **c** Simulated S-parameters for the different combinations of *c*_1_ and *c*_2_.

### Basic element for millimeter-wave self-multiplexing operation

In this section, the evolution of the two EMSIW cavity resonators are presented, which are utilized as the basic elements (BEs), for the design of self-multiplexing antenna for millimeter-wave spectrum. Figure 4a shows the electric field pattern of the hybrid TE_730_ mode, formed by the combination of the degenerate TE_370_ and TE_730_ modes. To realize the basic element 1 (BE1), the FMSIW cavity is bifurcated thrice using the magnetic-wall concept, resulting in the eighth-mode of the hybrid TE_730_ resonance. The hybrid TE_730_ mode is excited by feeding it through a microstrip feedline. Figure 4c illustrates the simulated S_CC_-parameters corresponding to various values of the design parameters *c*_1_ and *c*_2_ (shown in Fig. 4b). It can be observed from the figure that by selecting different values of *c*_1_ and *c*_2_ in BE1, different operating frequencies that lies between 28.7 GHz and 30.4 GHz can be achieved, which are subsequently utilized for the design of the lower millimeter-wave frequencies for self-multiplexing operation.

**Fig. 5:**
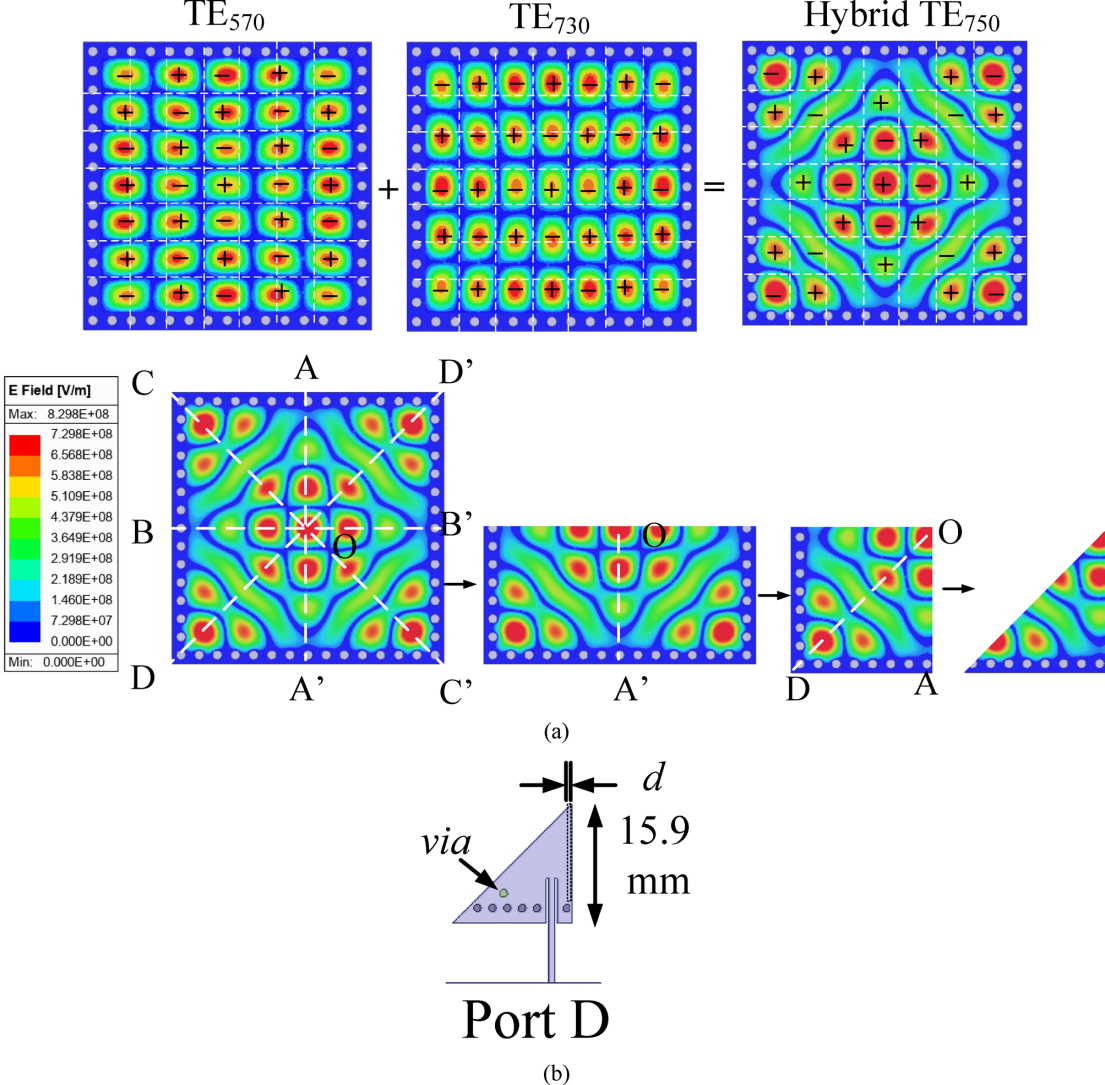
**a** Development stages of the EMSIW cavity resonator utilizing hybrid TE750 mode, **b** Microstrip-fed EMSIW cavity resonator.

**Fig. 6:**
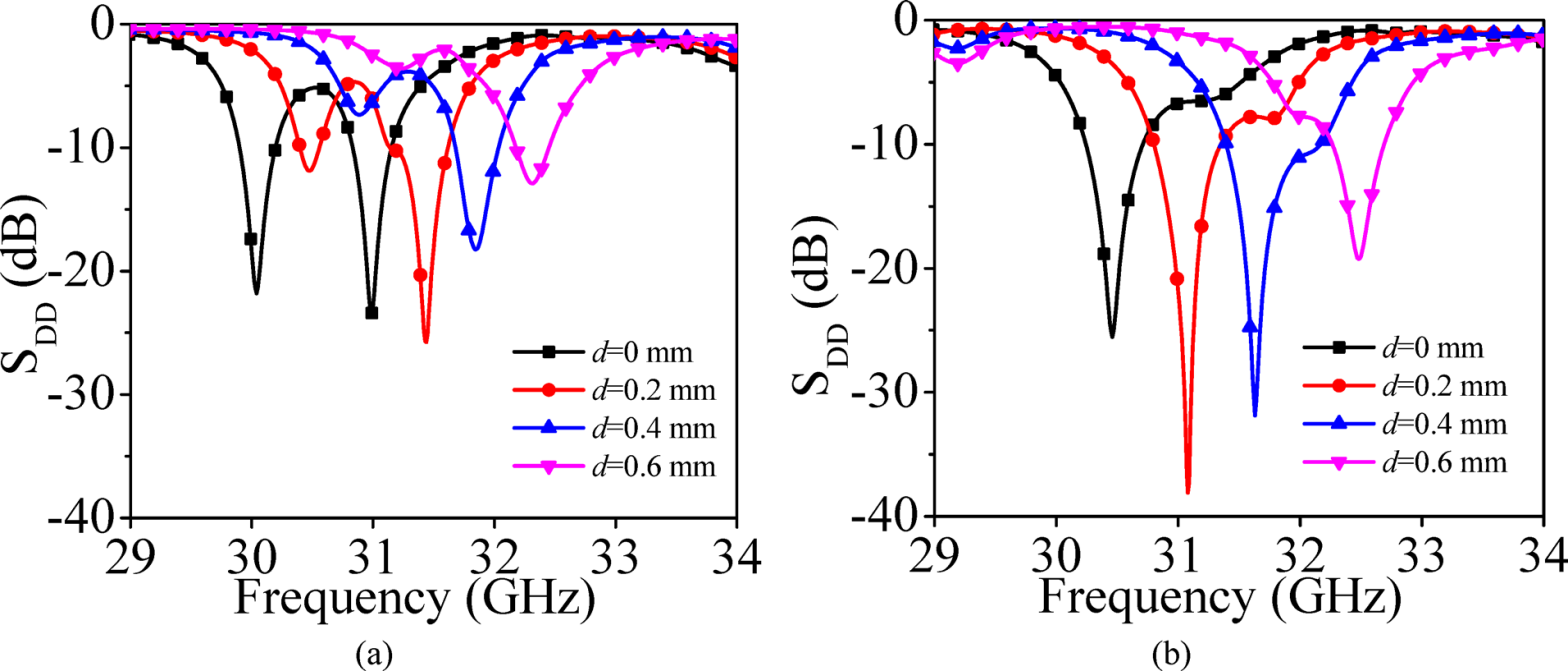
Simulated S-parameters for different combinations of design parameter *d*: **a** Without via, **b** With via (corresponding to the structure shown in (**b**)).

**Fig. 7:**
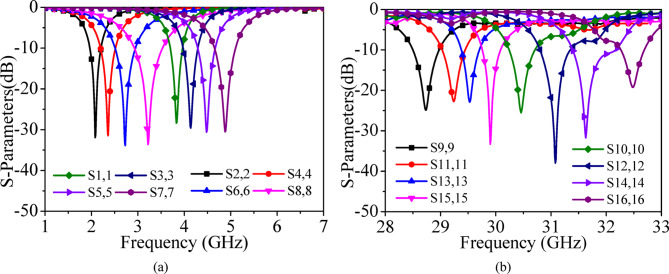
Simulated S_*ii*_-parameters of the designed integrated 16-port self-multiplexing: **a** For sub-6 GHz bands (S_*ii*_, *i* = 1, 2, 3…8), **b** For millimeter-wave frequency bands (S_*ii*_, *i* = 9, 10, 11,…16).

Figure 5a shows the electric field distribution of the hybrid TE_750_ mode, formed by the superposition of the degenerate TE_570_ and TE_750_ modes. Similarly, the basic element 2 (BE2) (Fig. 5b) is obtained by bifurcating the FMSIW cavity three times using the magnetic wall concept, thereby realizing the eighth-mode of the hybrid TE_750_ resonance, which is likewise excited through a microstrip feedline. Figure 6 illustrates the simulated S_DD_-parameters of BE2 for various values of the design parameter *d*, corresponding to the cases without and with vias, respectively. In the absence of the via, it can be observed from the figure that the excitation of two modes results in dual resonances at two distinct frequencies (Fig. 6a). A via is incorporated in the cavity to suppress higher-order resonances, resulting in a shift of the operating frequency to slightly higher values (Fig. 6b), thereby facilitating self-multiplexing operation in the upper millimeter-wave band from 30.5 to 32.5 GHz.

### Proposed 16-port integrated self-multiplexing antenna

Figure 1 shows the proposed 16-port integrated self-multiplexing antenna designed for operation in the sub-6 GHz and millimeter-wave spectrum. The proposed self-multiplexing antenna is realized by amalgamating the sub-6 GHz and millimeter-wave basic elements, as designed in the above sub-sections, onto the same substrate. The sub-6 GHz and millimeter-wave elements are placed alternately to efficiently utilize the available space while ensuring good inter-port isolation. Figure 7 presents the simulated reflection coefficients for the sub-6 GHz and millimeter-wave frequency bands. It can be observed from that the designed self-multiplexing antenna radiates at 3.85, 4.15, 4.50 and 4.85 GHz when Ports P_1_, P_3_, P_5_ and P_7_ are excited, and at 2.10, 2.35, 2.75, and 3.25 GHz when Ports P_2_, P_4_, P_6_ and P_8_ are excited. The designed self-multiplexing antenna demonstrates distinct radiation characteristics depending on the active port. Specifically, when Ports P_9_, P_11_, P_13_ and P_15_ are excited, the antenna radiates at frequencies 28.75, 29.25, 29.50 and 29.90 GHz, respectively. In contrast, when Ports P_10_, P_12_, P_14_ and P_16_ are activated, the antenna radiates at frequencies 30.50, 31, 32.65 and 32.50 GHz, respectively.

**Fig. 8:**
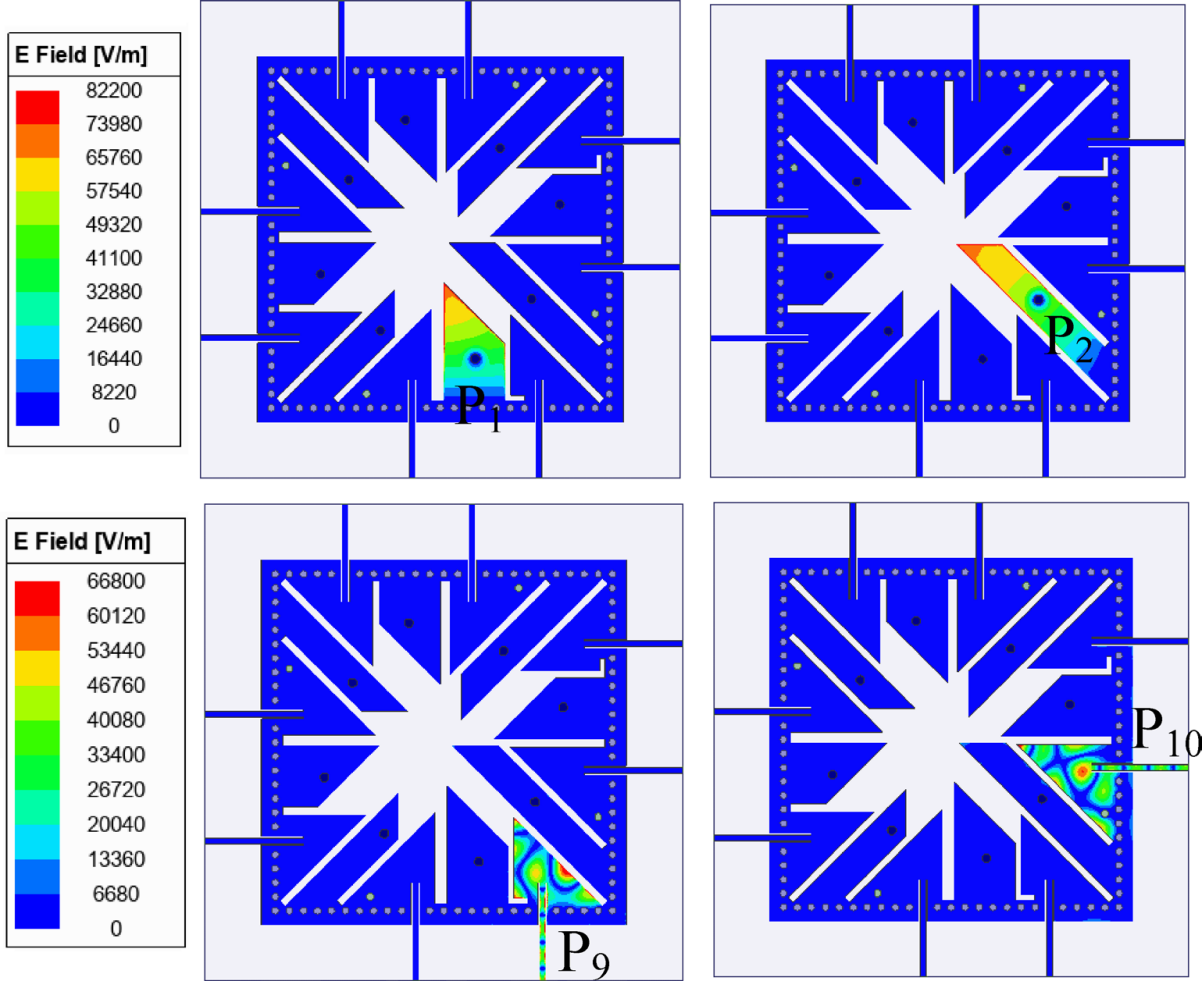
Electric field distribution corresponding to the excitations of Ports P_1_, P_2_, P_9_ and P_10_ at their respective operating frequencies.

In the sub-6 GHz band, the impedance bandwidths are 260, 270, 320, 330, 370, 380, 440 and 380 MHz for port excitations P_1_–P_8_, respectively. Whereas, in the millimeter-wave band, the impedance bandwidths are 470, 480, 420, 560, 360, 780, 340 and 430 MHz for port excitations P_9_–P_16_, respectively. These results confirm the antenna’s capability to simultaneously operate across multiple sub-6 GHz and millimeter-wave bands, demonstrating its suitability for 5G and beyond wireless communication systems. The designed antenna system can be efficiently utilized for self-multiplexing operations across various new radio (NR) services within the sub-6 GHz and millimeter-wave 5G spectrum^38,39^. In the sub-6 GHz band, the maximum channel bandwidth requirement is approximately 100 MHz, whereas in the millimeter-wave band, it extends up to around 400 MHz. The proposed antenna effectively satisfies these bandwidth demands, making it a strong candidate for integrated multiband 5G communication systems.

The inter-port isolation exceeding 40 dB is obtained in the sub-6 GHz frequency range, while isolation better than 20 dB is maintained in the millimeter-wave spectrum, thereby ensuring minimal mutual coupling between the ports. The isolation between the adjacent microwave elements (e.g., P_1_ and P_2_) is achieved not only through spatial diversity but also by employing distinct unit elements for each port. Specifically, EMSIW1 and EMSIW2, EMSIW cavity resonators, are individually modified to accommodate the millimeter-wave unit elements within the same substrate, enabling compact multiband integration while maintaining high inter-port isolation across the operating frequency spectrum. This structural variation minimizes the overlap of electromagnetic fields and effectively reduces mutual coupling. The isolation between other microwave unit elements (e.g., P_1_ and P_3_) can be attributed to their orthogonal placement and relatively large spatial separation, which further suppress coupling paths and enhance overall isolation. In the millimeter-wave band, isolation is primarily achieved through the orthogonal placement of unit elements (e.g., P_9_ and P_10_), while ports such as P_10_ and P_11_ employ different higher-order modes for radiation at millimeter-wave frequencies. The excitation of distinct modes, combined with large physical separation, minimizes electromagnetic interactions and ensures strong isolation among the millimeter-wave ports. Hence, the proposed antenna achieves excellent inter-port isolation through a combination of structural, modal and spatial isolation techniques. These strategies enable designed integrated self-multiplexing antenna suitable for advanced 5G and beyond communication systems.

**Fig. 9:**
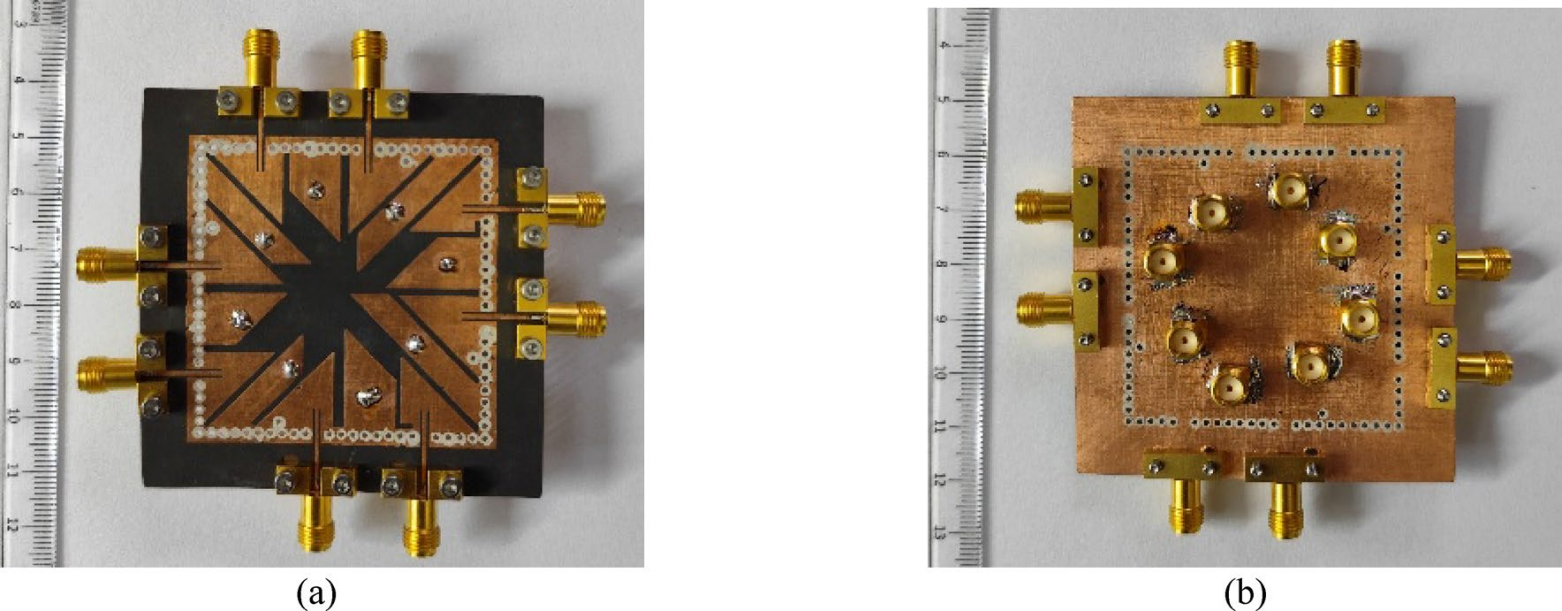
Fabricated prototype of the proposed integrated 16-port self-multiplexing antenna: **a** Top view, **b** Bottom view.

**Fig. 10:**
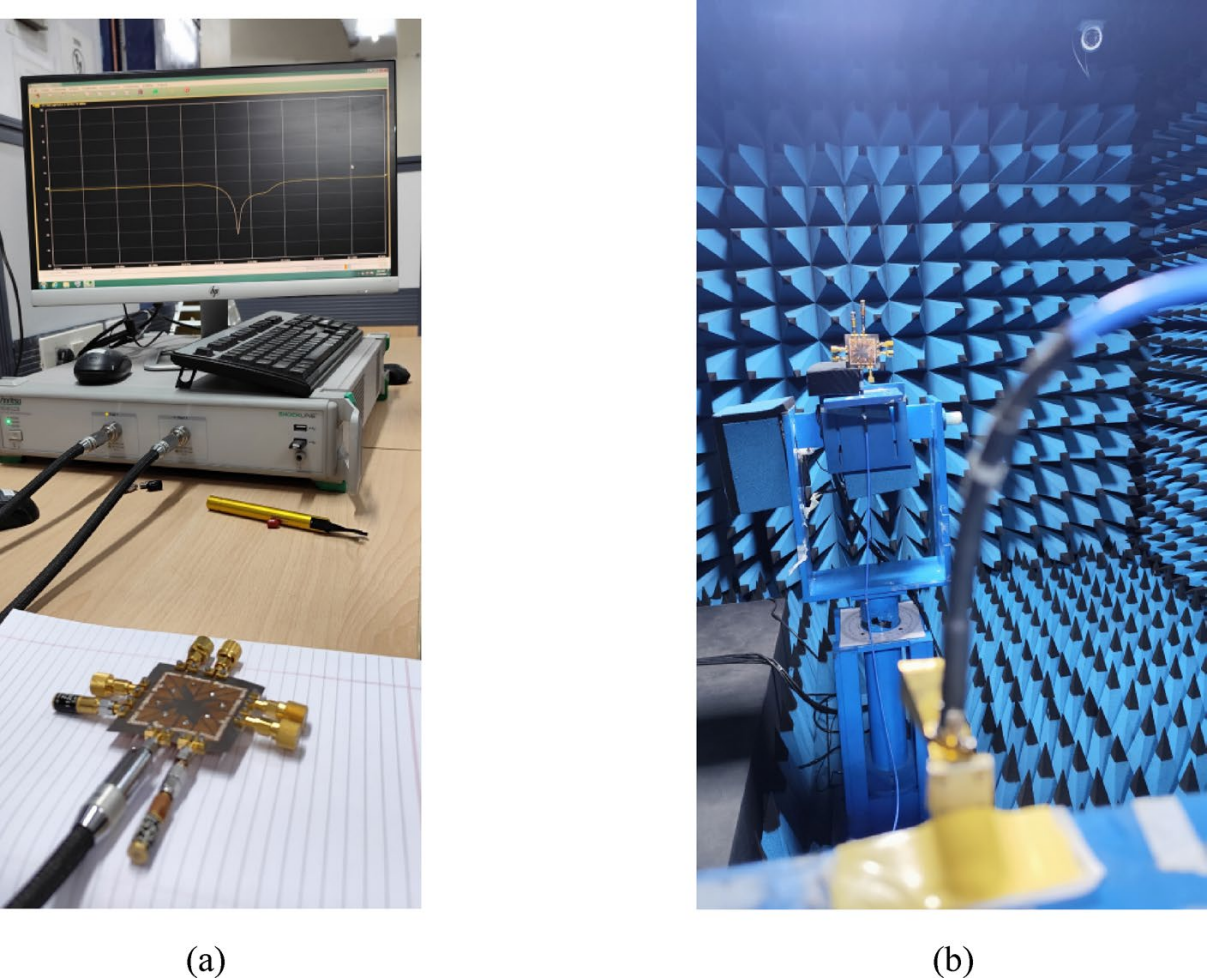
Measurement setup of the designed 16-port integrated self-multiplexing antenna: **a** S-parameter measurements, **b** Far-field radiation measurements.

To gain further insight into the minimal mutual coupling among the ports, the electric field distributions corresponding to the excitation of individual ports are plotted at their respective operating frequencies, as shown in Fig. 8. For brevity, only the excitations of Ports P_1_, P_2_, P_9_, and P_10_ are presented in this paper, as they are representative of the overall behaviour of the antenna. When one of the ports is excited, all the remaining ports are terminated with 50-Ω matched loads. The figure also illustrates that upon excitation of the respective ports, almost no fields are observed at the remaining ports, which confirms the excellent isolation characteristics of the design. It can also be observed that when Ports P_1_ and P_2_ are excited, they utilize the modified TE_110_ mode for radiation. Furthermore, the excitation of Ports P_9_ and P_10_ results in radiation through the hybrid TE_730_ and hybrid TE_750_ modes, respectively.

These behaviors highlight the versatility of the designed integrated antenna in achieving multiple radiation states through selective port excitations, thereby validating its self-multiplexing capability and ensuring effective operation across the designated frequency bands.

**Fig. 11:**
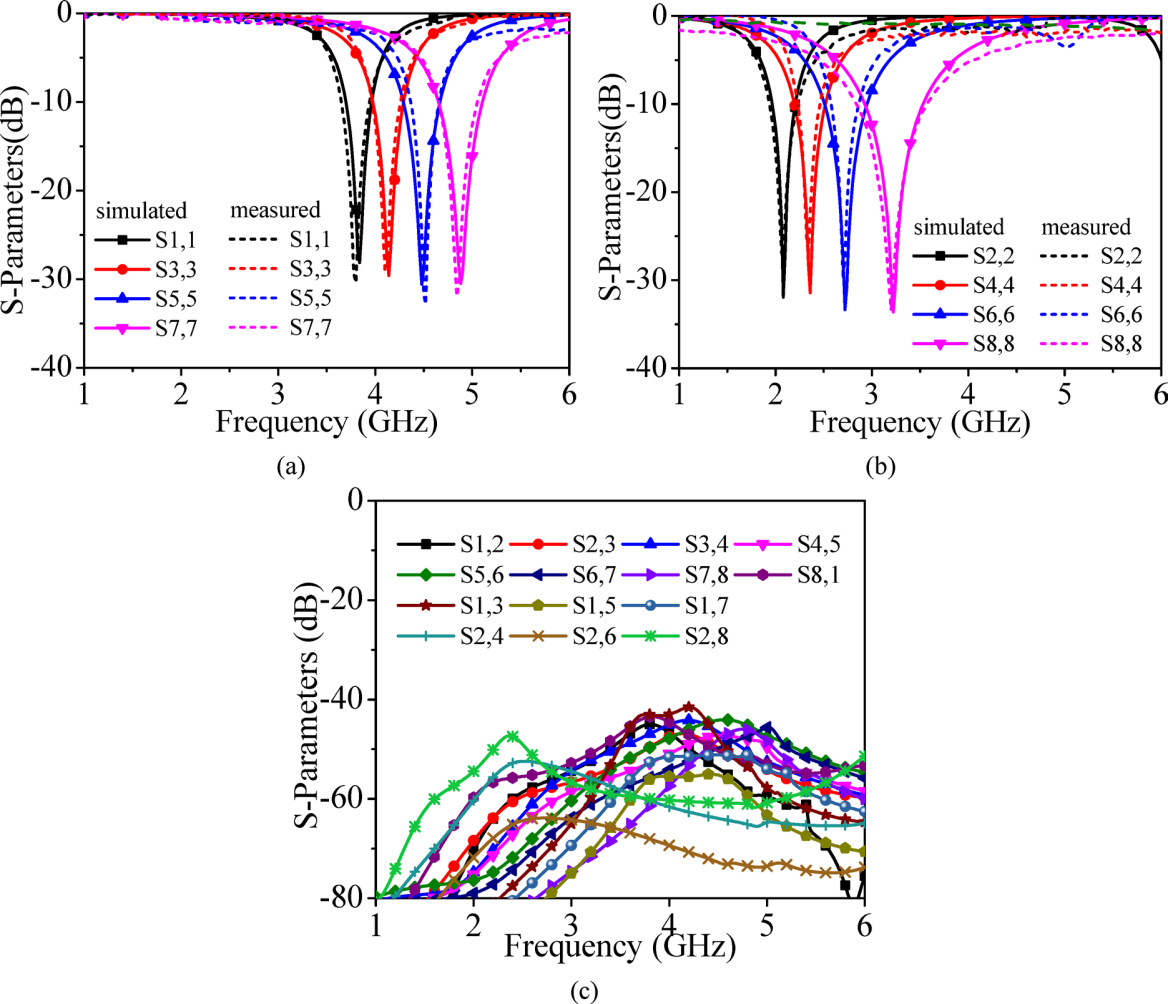
**a** Simulated and measured S_*ii*_-parameters (*i* = 1, 3, 5, 7), **b** Simulated and measured S_*ii*_-parameters (*i* = 2, 4, 6, 8), **c** Measured transmission coefficients.

## Results and discussion

This section presents the discussion of the measured results obtained from the fabricated prototype of the proposed self-multiplexing antenna system. A prototype of the designed antenna is fabricated using standard PCB fabrication techniques to experimentally validate the design methodology. The vias of the SIW cavity are drilled using an LPKF Protomat machine and filled with conductive silver epoxy through the plated-through-hole (PTH) process. Figure 9 illustrates the top and bottom views of the fabricated prototype of proposed integrated 16-port self-multiplexing antenna. For sub-6 GHz measurements, eight 32K101-400L5 coaxial SMA connectors are attached to Ports P_1_–P_8_, whereas for millimeter-wave frequencies, eight 02K243-40ME3 connectors are connected to Ports P_9_–P_16_ through microstrip feedlines. A 43.5 GHz Anritsu MS46322B vector network analyzer (VNA) is employed for S-parameter measurements, whereas the radiation patterns are characterized in an anechoic chamber as shown in Fig. 10.

**Fig. 12:**
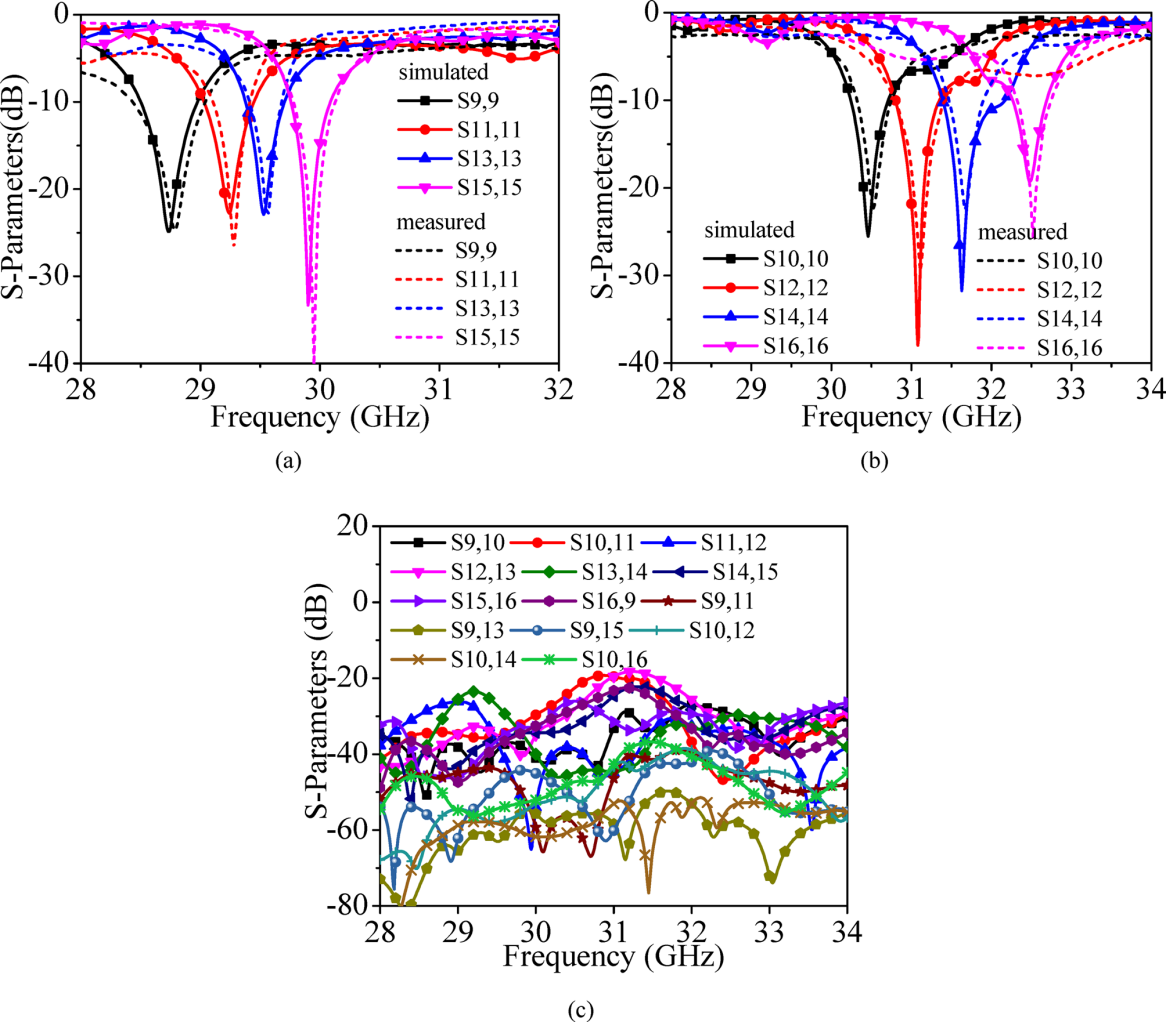
**a** Simulated and measured S_*ii*_ parameters (*i* = 9, 11, 13, 15), **b** Simulated and measured S_*ii*_-parameters (*i* = 10, 12, 14, 16), **c** Measured transmission coefficients.

Figure 11 presents the simulated and measured refection coefficients and measured transmission coefficients for Ports P_1_–P_8_, while Fig. 12 shows the corresponding results for Ports P_9_–P_16_. Under port excitations P_1_–P_8_, the proposed self-multiplexing antenna radiates at 2.10, 2.35, 2.75, 3.25, 3.85, 4.15, 4.50 and 4.85 GHz within the sub-6 GHz spectrum. In contrast, under Port excitations P_9_–P_16_, it operates at 28.75, 29.25, 29.50, 29.90, 30.50, 31, 31.65 and 32.50 GHz in the millimeter-wave spectrum. The designed self-multiplexing antenna exhibits measured inter-port isolations better than 40 dB in the sub-6 GHz spectrum and 20 dB in the millimeter-wave spectrum. The good inter-port isolations can be attributed towards the interleaving configuration of the sub-6 GHz and millimeter-wave antenna elements, which minimizes the mutual coupling. The discrepancies observed between simulated and measured responses can be attributed to connector and feeding losses, imperfections in the measurement environment and fabrication tolerances, where even minimal geometrical deviations can significantly influence performance at millimeter-wave frequencies. Nonetheless, the measured results exhibit good agreement with simulation, thereby confirming the reliability of the proposed antenna architecture.

**Fig. 13:**
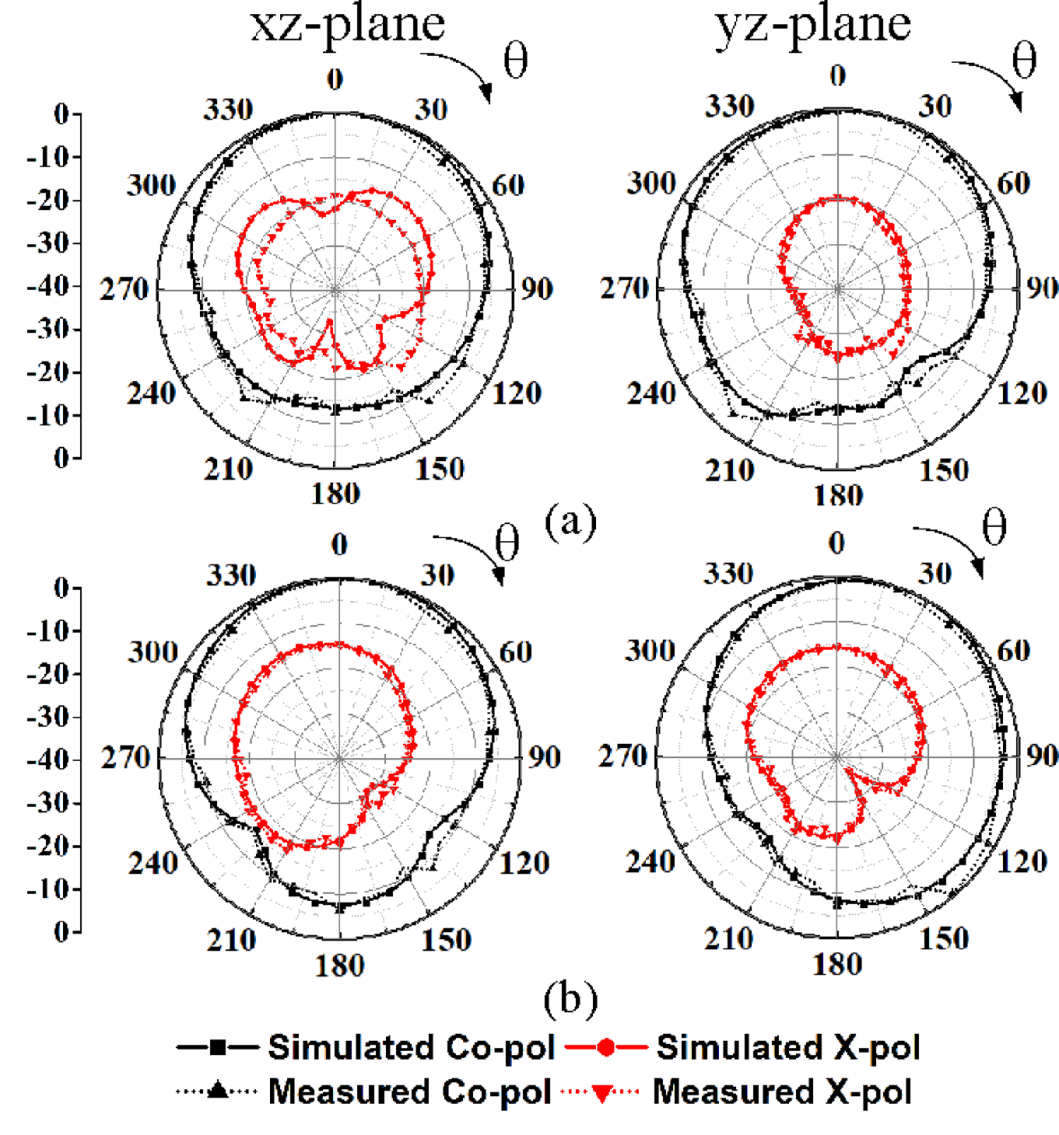
Simulated and measured radiation patterns: **a** Under port P_1_ excitation at 3.85 GHz, **b** Under port P_2_ excitation at 2.10 GHz.

**Fig. 14:**
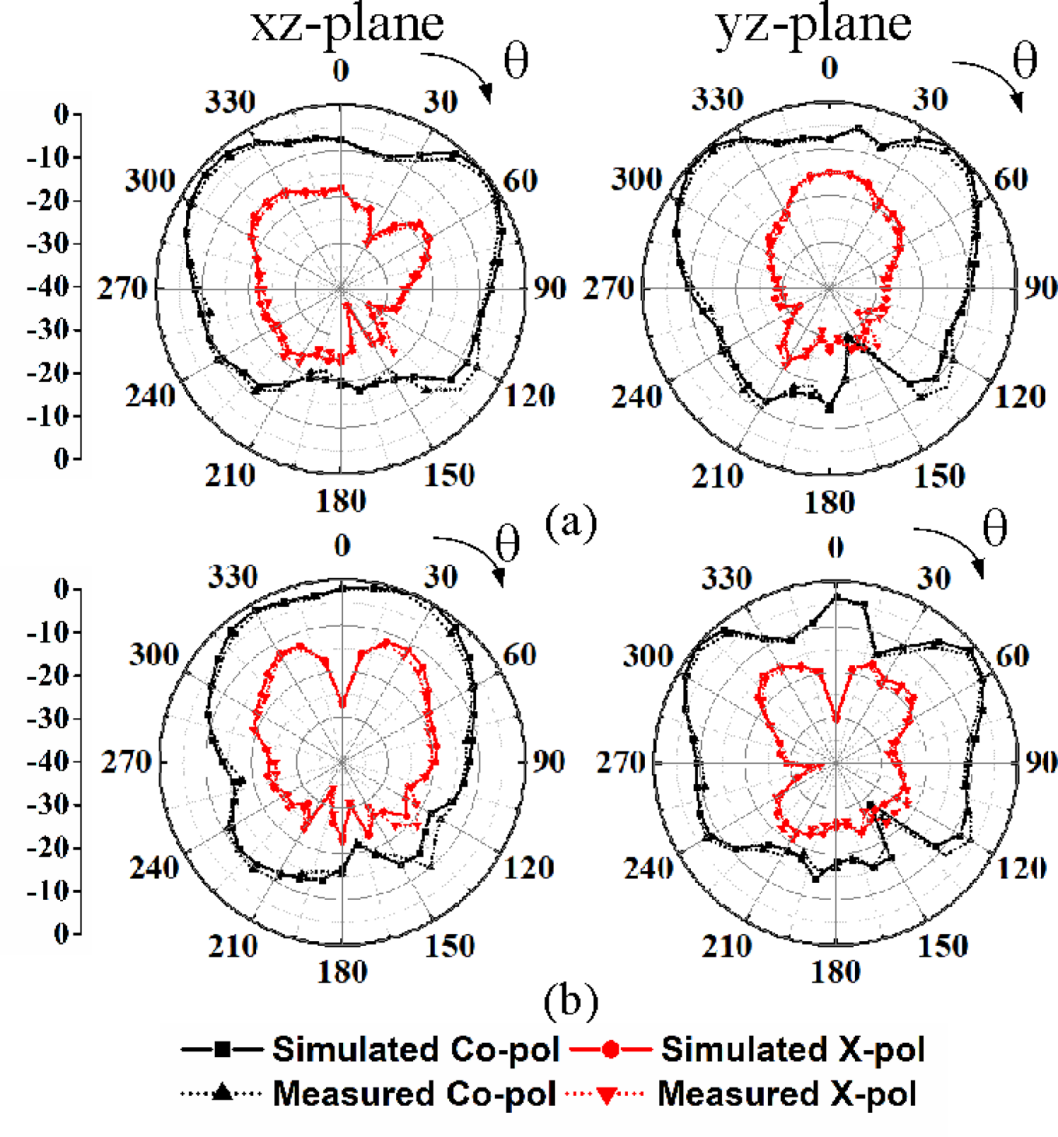
Simulated and measured radiation patterns: **a** Under port P_9_ excitation at 28.75 GHz, **b** Under port P_10_ excitation at 30.50 GHz.

Figures 13 and 14 present the simulated and measured radiation patterns of the proposed self-multiplexing antenna at sub-6 GHz and millimeter-wave frequencies, respectively. For conciseness, only the radiation patterns corresponding to Ports P_1_ and P_2_ excitations in the sub-6 GHz spectrum and Ports P_9_ and P_10_ excitations in the millimeter-wave spectrum are presented. At sub-6 GHz frequencies, the cross-polarization level is below −17 dB, whereas at millimeter-wave frequencies it remains below −15 dB in the direction of maximum radiations. The observed distinct radiation patterns at the sub-6 GHz and millimeter-wave frequency bands indicate that the proposed self-multiplexing antenna system can support a range of application scenarios in multiband 5G/6G communications. At the sub-6 GHz, the broader beamwidth and moderate directivity may be suitable for coverage enhancement, initial access and low-mobility user tracking. In contrast, at the millimeter-wave band, it can enable interference mitigation in dense urban environments. They offer enhanced pattern diversity, and lower spatial correlation, which are essential for robust link reliability in dynamic environments.

**Fig. 15:**
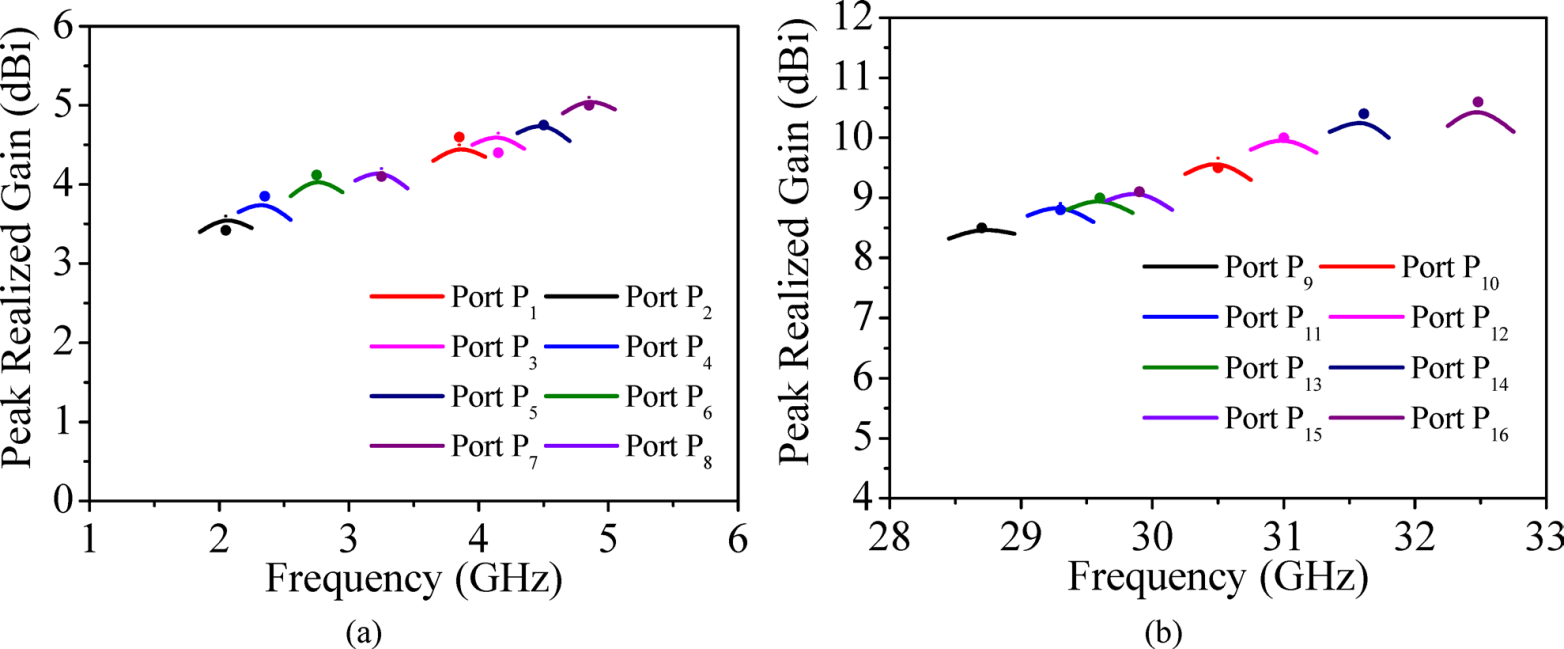
**a** Simulated and measured peak realized gain at sub-6 GHz, **b** Simulated and measured peak realized gain at millimeter-wave spectrum (solid line: simulated, dot: measured).

Figure 15 presents the simulated and measured peak realized gains of the proposed self-multiplexing antenna across different operating frequencies. The peak realized gains are 4.5, 3.6, 4.65, 3.8, 4.8, 4.1, 5.1 and 4.2 dBi corresponding to Ports P_1_ (3.85 GHz), P_2_ (2.10 GHz), P_3_ (4.15 GHz), P_4_ (2.35 GHz), P_5_ (4.50 GHz), P_6_ (2.75 GHz), P_7_ (4.85 GHz) and P_8_ (3.25 GHz), respectively. Whereas peak realized gains are 8.51, 9.65, 8.91, 10.05, 9.02, 10.35, 9.15 and 10.55 dBi under Ports P_9_ (at 28.75 GHz), P_10_ (at 30.5 GHz), P_11_ (at 29.25 GHz), P_12_ (at 31 GHz), P_13_ (at 29.50 GHz), P_14_ (at 31.65 GHz), P_15_ (at 29.95 GHz) and P_16_ (at 32.50 GHz) excitations, respectively. The higher gains achieved in the millimeter-wave spectrum are a consequence of exciting higher-order modes in EMSIW cavities. The radiation efficiencies exceed 80% in the sub-6 GHz band and 85% in the millimeter-wave spectrum.

**Fig. 16:**
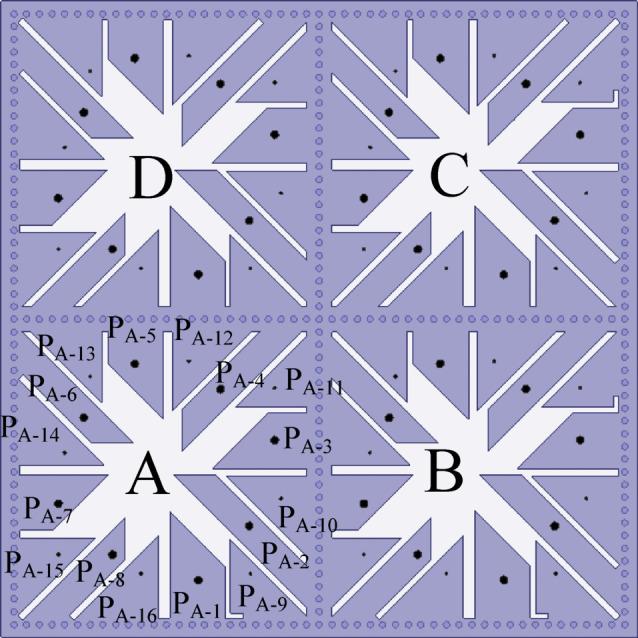
Architectural layout of the integrated self-multiplexing MIMO antenna.

The designed integrated 16-port sub-6 GHz and millimeter-wave self-multiplexing antenna can be utilized in advanced systems designed to meet the high data rate and low latency requirements for future generation communication networks. The usage of 16 ports in the proposed design is intentional and stems from the functional requirements of high capacity 5G wireless systems. A larger number of ports enables the antenna to generate multiple operating bands, which enable the efficient self-multiplexing operation for 5G IoT systems. Each port excites a distinct radiation mode, enabling efficient multiband operation while preserving low correlation and high isolation among the ports. This multi-port configuration also supports self-multiplexing operation, where multiple independent data streams can be transmitted simultaneously without requiring separate antenna structures. Therefore, the 16-port arrangement is not merely a structural choice but a performance driven requirement to meet the demands of advanced high data rate communication scenarios. It also plays a key role in the IoT, providing stable connections for a large number of devices simultaneously, which is essential for smart homes, industrial automation, and smart cities. In vehicular communications, it enables vehicle-to-everything (V2X) connectivity, enhancing traffic safety, autonomous driving, and infotainment systems^40–42^. The design methodology is versatile and can be implemented in diverse scenarios requiring communication across both the microwave and millimeter-wave spectrum.

### Integrated self-multiplexing MIMO antenna

Figure 16 illustrates the architectural layout of the 64-port MIMO configuration of the proposed integrated self-multiplexing antenna, which operates across 16 distinct frequency channels spanning both the sub-6 GHz and millimeter-wave spectrums. During the design of the MIMO configuration derived from the proposed integrated 16-port self-multiplexing antenna, all MIMO ports are coaxially fed. The MIMO antenna is implemented on the same substrate layer, namely RT/Duroid 5880. The electromagnetic characteristics of the MIMO configuration are analyzed using Ansys HFSS (v. 22). In the MIMO configuration, four square SIW cavities, denoted as cavity A, B, C and D, are employed as shown in Fig. 16. Each cavity integrates 16 ports, where the first eight ports operate in the sub-6 GHz band and the remaining eight ports correspond to millimeter-wave frequencies. Ports within the cavities are designated P_A-1_–P_A-16_, P_B-1_–P_B-16_, P_C-1_–P_C-16_ and P_D-1_–P_D-16_. Ports sharing the same index (e.g., P_A-X_, P_B-X_, P_C-X_, P_D-X_ for *x* = 1, 2, …16) exhibit identical operating frequencies. As discussed earlier, the isolation among the ports located within each cavity is better than 20 dB in both frequency bands. In contrast, the isolation among ports belonging to different cavities exceeds 30 dB, owing to the presence of the via sidewalls, which confine the electromagnetic fields within each cavity. Thus, the designed antenna has the capability to be scaled to higher order MIMO antenna in order to demonstrate its applicability in real 5G systems.

## Conclusion

This article introduced a compact 16-port integrated self-multiplexing antenna for sub-6 GHz and millimeter-wave 5G frequency bands with excellent inter-port isolation. The designed self-multiplexing antenna possesses dual-spectrum operation in sub-6 GHz and millimeter-wave frequency bands. The TE_110_ mode is excited in the modified EMSIW cavity resonators for radiation within the microwave spectrum, while hybrid TE_730_ and TE_750_ modes of the EMSIW cavity resonators are excited for operation in the millimeter-wave frequency spectrum. The first eight ports radiate at 2.10, 2.35, 2.75, 3.25, 3.85, 4.15, 4.50 and 4.85 GHz frequencies of the sub-6 GHz spectrum through TE_110_ mode, while the remaining eight ports operates at 28.75, 29.25, 29.55, 29.95, 30.50, 31, 31.65 and 32.50 GHz frequencies of the millimeter-wave spectrum through hybrid higher order modes. It possesses impedance bandwidth ranging from 260 to 440 MHz for Ports P_1_–P_8_ in the microwave band, and from 340 to 780 MHz for Ports P_9_–P_16_ in the millimeter-wave band. The peak realized gains corresponding to Ports P_1_–P_8_ are 4.5, 3.6, 4.65, 3.8, 4.8, 4.1, 5.1 and 4.2 dBi, respectively, whereas those for Ports P_9_–P_16_ are 8.51, 9.65, 8.91, 10.05, 9.02, 10.35, 9.15 and 10.55 dBi under the excitation of the respective ports. High isolation (≥ 20 dB) among the antenna elements is achieved, which can be attributed towards the interleaved placement of the sub-6 GHz and millimeter wave basic elements inside a square SIW cavity. The design antenna system has an overall footprint of 0.425$\:{\lambda\:}_{g}^{2}$, where $\:{\lambda\:}_{g}$ corresponds to guided wavelength at the lowest operating frequency of 2.1 GHz. With its compact form factor and good performance, the proposed integrated self-multiplexing antenna holds significant potential for communication systems in both sub-6 GHz and millimeter-wave 5G frequency ranges.

## Data Availability

The datasets used and/or analyzed during the current study available from the corresponding author on reasonable request.
